# Synaptic vesicle tethering and the CaV2.2 distal C-terminal

**DOI:** 10.3389/fncel.2014.00071

**Published:** 2014-03-07

**Authors:** Fiona K. Wong, Arup R. Nath, Robert H. C. Chen, Sabiha R. Gardezi, Qi Li, Elise F. Stanley

**Affiliations:** Laboratory of Synaptic Transmission, Toronto Western Research InstituteToronto, ON, Canada

**Keywords:** presynaptic, calcium channel, synaptic vesicle, tether, SV-PD, RIM binding protein, cryoloading, PDZ

## Abstract

Evidence that synaptic vesicles (SVs) can be gated by a single voltage sensitive calcium channel (CaV2.2) predict a molecular linking mechanism or “tether” (Stanley, 1993). Recent studies have proposed that the SV binds to the distal C-terminal on the CaV2.2 calcium channel (Kaeser et al., 2011; Wong et al., 2013) while genetic analysis proposed a double tether mechanism via RIM: directly to the C terminus PDZ ligand domain or indirectly via a more proximal proline rich site (Kaeser et al., 2011). Using a novel* in vitro *SV pull down binding assay, we reported that SVs bind to a fusion protein comprising the C-terminal distal third (C3, aa 2137–2357; Wong et al., 2013). Here we limit the binding site further to the last 58 aa, beyond the proline rich site, by the absence of SV capture by a truncated C3 fusion protein (aa 2137–2299). To test PDZ-dependent binding we generated two C terminus-mutant C3 fusion proteins and a mimetic blocking peptide (H-WC, aa 2349–2357) and validated these by elimination of MINT-1 or RIM binding. Persistence of SV capture with all three fusion proteins or with the full length C3 protein but in the presence of blocking peptide, demonstrated that SVs can bind to the distal C-terminal via a PDZ-independent mechanism. These results were supported *in situ* by normal SV turnover in H-WC-loaded synaptosomes, as assayed by a novel peptide cryoloading method. Thus, SVs tether to the CaV2.2 C-terminal within a 49 aa region immediately prior to the terminus PDZ ligand domain. Long tethers that could reflect extended C termini were imaged by electron microscopy of synaptosome ghosts. To fully account for SV tethering we propose a model where SVs are initially captured, or “grabbed,” from the cytoplasm by a binding site on the distal region of the channel C-terminal and are then retracted to be “locked” close to the channel by a second attachment mechanism in preparation for single channel domain gating.

## INTRODUCTION

At fast synapses neurotransmitters are released by the fusion and discharge of synaptic vesicles (SV) at transmitter release sites within the active zone (AZ). Action potentials that invade the terminal open voltage gated calcium channels (CaV) and admit Ca^2^^+^ which diffuses to bind to a SV calcium sensor to trigger fusion. Based on the finding that a single CaV can gate the fusion of an SV, our previous work predicted that the calcium sensor must be within the high-Ca^2^^+^ domain of the SV and, hence, physically attached or “tethered” to the channel (Stanley, 1993). While initially contested, this idea has recently gained general acceptance (Mulligan et al., 2001; Wachman et al., 2004; Gentile and Stanley, 2005; Shahrezaei et al., 2006; Weber et al., 2010; Jarsky et al., 2010; Eggermann et al., 2011; Matveev et al., 2011; Sheng et al., 2012; Atlas, 2013; Tarr et al., 2013).

Several mechanisms of SV tethering by CaVs have been proposed and these fall into two main classes: indirectly via surface membrane docking proteins (Catterall, 1999), or directly via a cytoplasmic link (Wong and Stanley, 2010; Kaeser et al., 2011; Wong et al., 2013). The reduction in transmitter release noted in heterozygote* leaner* mice, that express a CaV2.1 truncated C-terminal (Kaja et al., 2008), may have been an early hint that this region of presynaptic CaVs plays a role in transmitter release. CaV2.2 type channels, in particular the long C-terminal splice-variant (Maximov and Bezprozvanny, 2002; Khanna et al., 2006b), are well established to gate transmitter release at presynaptic terminals. The possibility that SVs tether to the long-splice region has recently sparked particular interest (Kaeser et al., 2011; Wong et al., 2013). A molecular model has been proposed in which “Rab3 interacting molecule” [RIM; which interacts with a variety of Rab species (Fukuda, 2003)] binds to the SV via its namesake and serves as a bridge to the channel via two interactions. In the first of these the PDZ domain in RIM binds directly to a DxWC PDZ ligand motif at the C-terminus. The proposed second link was indirect: RIM links to the channel via RIM-binding-protein (RBP; Hibino et al., 2002) and attaches to a proline-rich PxxP motif (termed here the P^**^P domain) in the distal third of the C-terminal (Kaeser et al., 2011). Using a novel “SV pull down” (SV-PD) *in vitro* assay, we have recently demonstrated that native CaV2.2 can capture SVs and that this capture can be replicated with a fusion protein mimicking the distal third of the C-terminal, amino acids (aa) 2138 to 2357 (in chick), a region we term C3. Our quantitative immunocytochemical analysis [Intensity Correlation Analysis, (Li et al., 2004)] supported the idea that that CaV2.2 and RIM co-vary at presynaptic transmitter release sites (Khanna et al., 2006a). However, the failure to detect a CaV2.2-RIM complex by biochemical analysis suggests that these proteins are parts of two independent, but possibly transiently interacting, complexes (Khanna et al., 2006a; Wong and Stanley, 2010; Wong et al., 2013) and is at odds with the current tether molecular model. 

We set out to explore C3-to-SV binding by SV-PD and standard biochemical methods using SVs purified from chick brain synaptosomes (SSMs), channel C-terminal constructs and synthetic blocking peptides. These were complemented by novel methods of “SSM-ghost electron microscopy” (EM) to image tether-like structures, and peptide “cryoloading” (Nath et al., 2014) to test binding site predictions on SV recycling in intact, functional SSMs. We provide additional support for SV tethering by the C-terminal and conclude that this involves a novel, but not yet localized, binding site within a 49 aa region, proximal to the tip PDZ-ligand domain. Since the predicted length of the extended C-terminal is too long to account for the required close association of the channel to the docked vesicle (~25 nm; Stanley, 1993; Weber et al., 2010), we suggest that while this tether may account for the capture of SV from the cytoplasm, tethering is completed by subsequent additional channel-SV interactions.

## MATERIALS AND METHODS

### SYNAPTOSOME AND SYNAPTIC VESICLE FRACTIONATION AND SOLUBILIZATION

These have been described in detail (Juhaszova et al., 2000; Wong and Stanley, 2010; Gardezi et al., 2013; Wong et al., 2013). Key preparation buffers were: homogenization buffer (HB), 0.32 M sucrose, 10 mM HEPES, 2 mM EDTA, pH 7.4; HEPES lysis buffer, 50 mM HEPES, 2 mM EDTA, pH 7.4; and modified radioimmunoprecipitation assay solubilization buffer (RIPA), 50 mM Tris-HCl, 150 mM NaCl, 1% NP-40, 0.5% Na^+^ deoxycholate, 1 mM EDTA, pH 8.4)

### ANTIBODIES

Antibodies used in this study and concentrations used for blotting are listed in **Table 1**.

**Table 1:** Antibodies.

Antibody	Target	Mono/polyclonal	Source	WB dilution
FLAG	FLAG	m p#	Sigma-Aldrich Co. Cell signaling	1:4000
GST	GST	m	Santa Cruz Biotechnology	1:4000
L4569	Long splice variant of Cav2.2 C-terminal	p	Stanley lab (Khanna et al., 2006b)	–
MINT-1	MINT-1	m	BD Biosciences	1:500
RIM1 (mRIM)	RIM (RIM1 and 2)*	m	BD Biosciences	1:1000
RIM2 (pRIM2)	RIM (RIM1 and 2)*	p	Synaptic Systems GMBH	1:2000
Strep	Strep	m	IBA	1:2000
SV2A	SV2A	m	Synaptic Systems GMBH clone 171G0	1:1000
ASV30	Synaptotagmin	m	Abcam Inc.	1:1000
VAMP2	VAMP2	p	Enzo Life Sciences	1:2000

*See characterization in Wong and Stanley (2010). Note that pRIM2 is termed RIM1, 2 in that paper. #FLAG antibodies were used interchangeably.

### GENERATION OF FUSION PROTEINS

For C3_Strep_ (see: Gardezi et al., 2013), a PCR fragment of the CaV2.2 long splice (*cdB1*) variant (aa 2138–2357) was subcloned into pPr-IBA (IBA) expression vector with the Twin-Strep-tag (this was previously named “One-Strep”) at the N-terminus [sequence: SA-WSHPQFEK(GGGS)2GGSAWSHPQFEK (IBA)]. The GST-tagged fusion protein C3_Prox_ (aa 2138–2299) PCR fragment was subcloned into a pGEX-KG (GE Healthcare) vector and GST-FLAG-tagged proteins (C3_WildF_ and C3_MutantF_, aa 2138–2357) into a pGEX-KG expression vector with a sub-cloned FLAG tag. The DNA sequence in frame was confirmed by sequencing after transformation into DH5α competent cells (Invitrogen). C3_Strep_ was used on bead whereas GST fusion proteins were eluted using 20 mM reduced Glutathione (Bioshop), in 50 mM Tris-HCl pH 8.0. GST fusion proteins were concentrated using a 10K Microcon and stored in PBS (GIBCO; Life technologies).

### BIOCHEMICAL ASSAYS

Standard Western blots and pull-down assays were as described (Wong and Stanley, 2010; Gardezi et al., 2013).

### SYNAPTIC VESICLE BINDING ASSAY

The novel SV-PD method is described in detail in a recent report (Wong et al., 2013). Briefly, we immobilized a bait fusion protein on a precipitation bead as for standard PD but exposed it to a suspension of purified SVs in the detergent free, HB buffer with free Ca^2^^+^ clamped to 10 nM (5 mM EGTA plus CaCl_2_ calculated using MaxChelator, maxchelator.stanford.edu) throughout. The beads were then washed and proteins solubilized for Western blot analysis. To test for SV-PD we selected integral proteins that could be used as markers for SV capture: SV2A, synaptotagmin (STG) and vesicle-associated membrane protein-2 (herein VAMP). Positive SV-PD capture was concluded if bands for two of these integral SV proteins were obviously darker than the vector control. Densitometry was also used to quantify individual protein bands for a particular data set, as described followed by statistical analysis (see below) for whether the mean was significantly above zero.

### AMINO ACID MIMETIC PEPTIDES

We synthesized mimetic or control peptides for the P^*^^*^P region (PQTP in chick) and the C terminus PDZ ligand-domain region (DDWC) that included: RQLPQTPL (P^*^^*^P, aa 2210–2217); HEADEDDWC (H-WC; aa 2349–2357); DDWA (aa 2354–2357), and HEADE (aa 2349–2353; Hospital for Sick Children Advanced Protein Technology Peptide Synthesis Facility). Peptides were made as a 10 mM stock solution in HB or RIPA buffer, and 1–1.2 mM peptide was added to each sample for pull-down experiments.

### SYNAPTOSOME PEPTIDE CRYOLOADING AND SV RECYCLING ASSAY

This method is described in detail in a recent paper (Nath et al., 2014). Imaging was carried out on a Zeiss Axioplan2 with a 63×, 1.4 NA objective.

### SYNAPTOSOME GHOST ELECTRON MICROSCOPY

Synaptosomes used for SSM ghost preparation were prepared in HB with 1 mM EGTA in place of 2 mM EDTA. SSM ghosts were prepared by hypotonic lysis of SSMs as for SV isolation except using a lysis buffer in which free Ca^2^^+^ is buffered to 0.1 μM (EGTA/Ca^2^^+^ ratio calculated using MaxChelator). The ghosts were retrieved by a 30 min 20,000 × *g* spin and were resuspended in a 0.2 M sucrose HB with 1 mM EGTA. The suspension was then loaded onto a second (0.4 M/0.6 M/0.8 M/1.0 M sucrose) gradient and the ghosts were collected from the 0.8/1.0 M interface. Ghosts were pelleted at 16,000 × *g* for 1 h. The pellet was fixed, dehydrated, embedded, and sectioned for EM as described (Nath et al., 2014). Imaging was carried out on HT7000, HT7500, or HT7700 electron microscopes (Cell and Systems Biology, University of Toronto, or University of Toronto at Scarborough imaging facilities).

### IMMUNOBLOT ANALYSIS AND STATISTICS

The study of cell membrane components in detergent-free conditions is prone to non-specific binding and high background protein staining. This challenge was dealt with primarily by running paired GST or strep vector (Strep_V_), as appropriate, controls for all test lanes and rejecting protein bands associated with contaminated control lanes (see Wong and Stanley, 2010; Wong et al., 2013 for criteria). We used several analysis methods. Protein bands were quantified by densitometry of immunoblot films as described (Wong and Stanley, 2010; Wong et al., 2013) and we tested for significant difference of the mean from zero (p_t_ = 0). For SV-PD experiments, however, the primary question is discrete: whether or not the SV protein was detected, not its amplitude. To test this question we used a binomial approach. Individual immunoblots were scored as positive for the capture of a particular SV protein marker if the test band was unequivocally darker than its control, GST- or Strep_V_-only lane. A large data set with the control, native-sequence fusion protein, C3_Strep_, was used to predict the expected SV capture probability. This was then used to test the capture frequency observed with modified conditions (e.g., mutant fusion protein) by binomial analysis (see text and **Table 2**). We scored individual SV-PD experiments as positive for SV capture based on the detection of at least two of our three integral SV protein markers (SV2, STG, VAMP) and tested if these were significantly different from the capture rate with C3_Strep_ by binomial analysis.

**Table 2:** SV-PD binomial analysis.

**(A)**					**(B)**
	**SV2**	**STG**	**VAMP2**	**RIM**	**C3_Strep_*p* = 0.86**	**SV-PD**	***p***
C3_Strep _*n/N*	10/13	13/14	13/19	19/22	C3_Prox_	1/4	**<0.02**
*p*	0.769	0.929	0.684	0.826
C3_Prox_	1/4	0/4	1/5	0/5	C3_MutantF_	5/5	>0.1
	**<0.05**	**<0.01**	**<0.02**	**<0.01**
C3_MutantF_	5/5	5/5	3/4	3/5	C3_W__ildf_	5/5	>0.1
	>0.1	>0.1	>0.1	>0.1
C3_WildF_	5/5	5/5	2/4	3/5	C3_S__trep_ + H-WC	4/4	>0.1
	>0.1	>0.1	>0.1	>0.1
H-WC	5/5	4/5	3/3	2/3	C3_Strep_ + P^*^^*^P	3/4	>0.1
	>0.1	>0.1	>0.1	>0.1

***(A)** The top row is the fraction of experiments with a positive protein capture with C3_Strep_*(n) *over the number of experiments analyzed (*N*) and the calculated ratio, *p*, was used as the predicted success rate. The *n/N *fraction is given for each protein with each test fusion protein. Statistical difference was calculated by the binomial distribution and significantly different ratios are indicated by bold text. **(B)***n/N *fractions for successful SV-PD (as defined for each experiment as capture of two or more marker SV proteins) is given for each protein. Statistical difference was calculated using the binomial distribution against the predicted capture using C3_Strep_.*

## RESULTS

### SVS BIND TO THE DISTAL TIP OF THE CHANNEL C-TERMINAL

**FIGURE 1:**
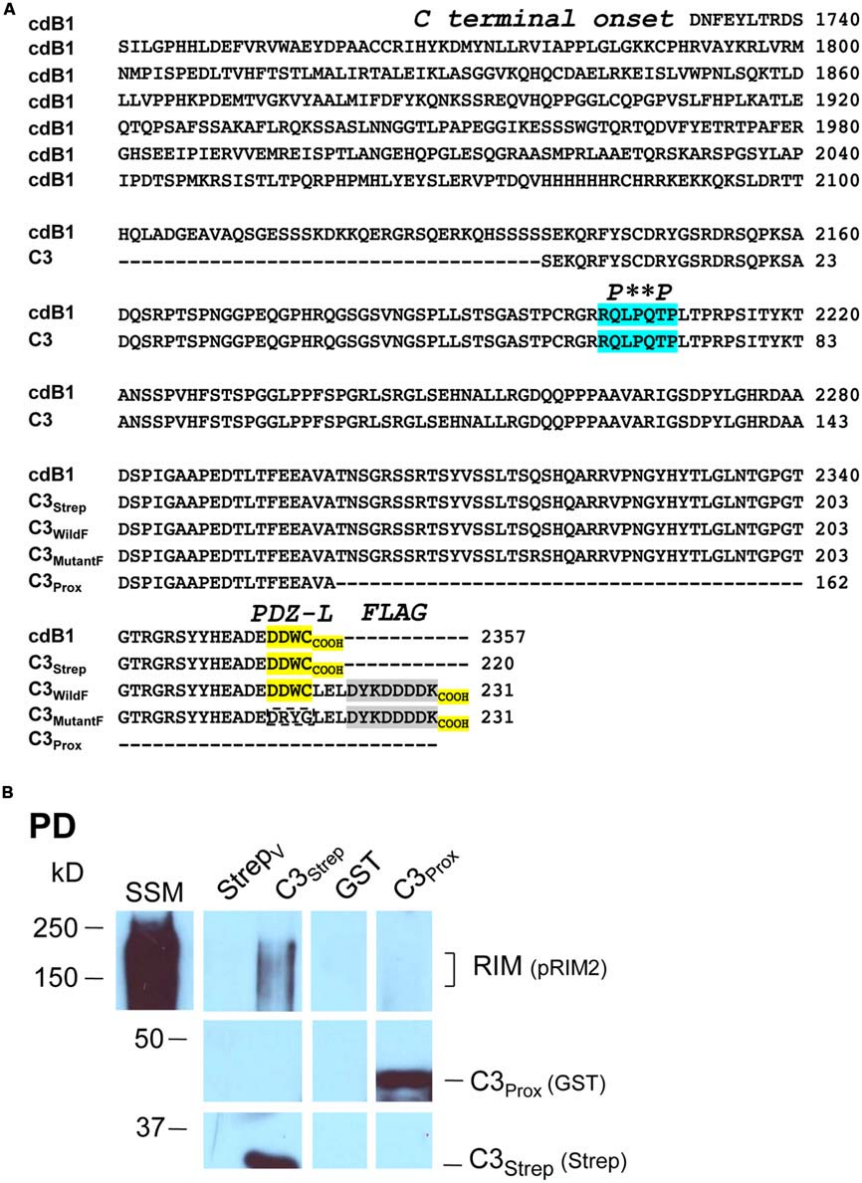
**Fusion proteins. (A)** Amino acid residues for native (cdB1) and C3 fusion protein constructs. Four constructs are shown: C3_Strep_, C3_WildF_, C3_MutantF_, and C3_Prox_ with their common proximal region labeled C3. The proposed RBP P^*^^*^P binding domain (*blue*), the terminal PDZ ligand domains (*yellow*) and the FLAG tag attached to C3_WildF_ and C3_MutantF_ (*gray*) are highlighted. In addition to the added FLAG tag, the DDWC PDZ ligand domain is mutated to DRYG in C3_MutantF_ (*dashed box*). * any amino acid. **(B)** C3_Strep_, but not C3_Prox_, pulls down RIM from SSM membrane lysate. WB, Western blot; Strep_V_, strep vector alone; probing antibodies are indicated in brackets.

**FIGURE 2:**
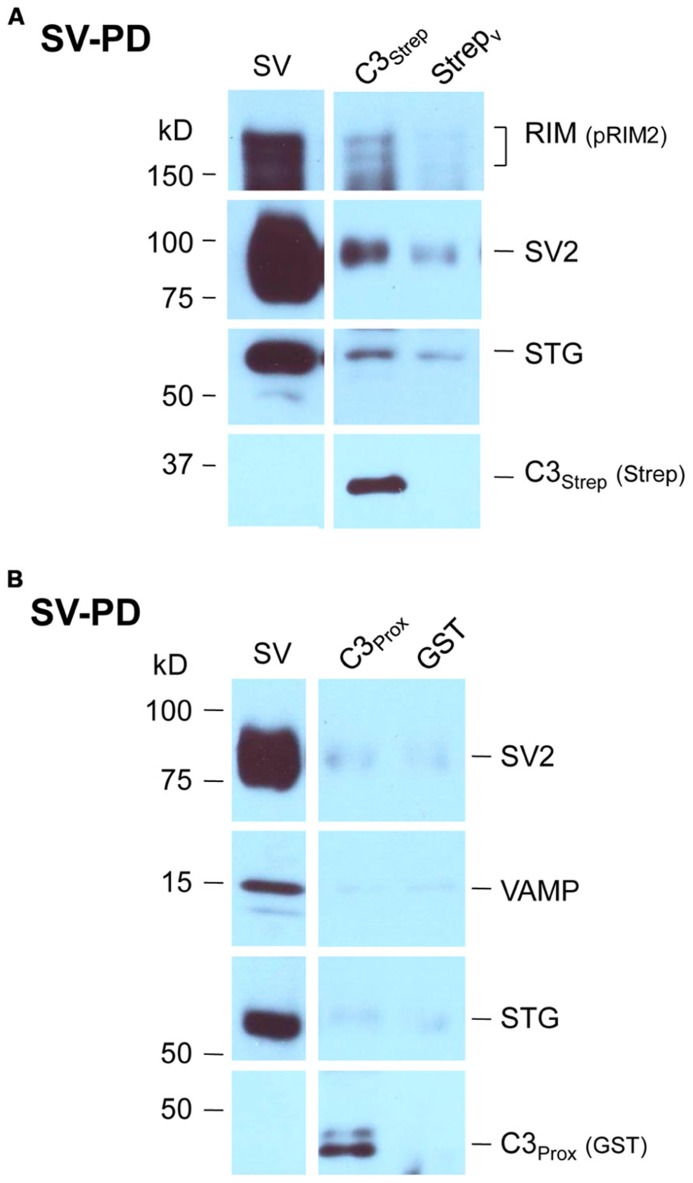
**SV pull down requires the distal region of the C-terminal. (A)** Bead-immobilized C3_Strep_ was exposed to a suspension of purified SVs. SV capture was assayed by standard WB for key marker proteins (see text). **(B) **As in (**A)**, but using immobilized C3_Prox_. SV capture failed, as indicated by the absence of integral protein markers.

We recently used the SV-PD assay to demonstrate that SVs can be captured by a fusion protein (C3_Strep_) comprising the distal 220 aa of the channel C-terminal (**Figure 1A**; Wong et al., 2013) and, hence, that this region contains a potential SV tether site. To further restrict the location of the SV binding site within the C3 sequence we created a second fusion protein, C3_Prox_, that lacks the distal 58 aa (**Figure 1A**; Gardezi et al., 2013). As expected, and in contrast to C3_Strep_, this fusion protein failed to pull down the modular adaptor protein MINT-1 (Gardezi et al., 2013) and RIM (**Figure 1B**)**which are known to bind to the PDZ ligand domain (Maximov et al., 1999). We next used SV-PD to test if C3_Prox_ could capture SVs. The fusion protein was immobilized on standard glutathione beads, exposed to purified SVs in a detergent-free buffer (HB), and SV capture was assessed by Western blot for signature integral proteins (see Materials and Methods). In contrast to the full-length C3 fusion protein, C3_Strep_ (**Figure 2A**; Wong et al., 2013) SV-PD was not detected with C3_Prox_ using our criteria (**Figure 2B**; statistical analysis of band densities: STG 0.02 ± 0.01 *U*, *N* = 4, p_t_ = 0 > 0.1; SV2 0.01 ± 0.01 *U*, *N* = 3, p_t_ = 0 > 0.1; VAMP 0.03 ± 0.03 *U*, *N* = 4, p_t_ = 0 > 0.1; *U* = dimensionless intensity units). Since the primary question in this study was whether SVs were captured and not how much protein was recovered, we scored each experiment as positive or negative for each protein and then used a discrete statistical analysis method. We used our large dataset with C3_Strep_ to establish a predicted capture frequency for the normal distal C-terminal: *n/N*, where *n* is the number of experiments with positive recovery and *N* the total number of experiments. Binomial analysis showed that the SV capture with C3_Prox_ was significantly less than with C3_Strep_, based either on individual SV marker proteins (**Table 2A**) or SV-PD (**Table 2B**; see Materials and Methods). Thus, we concluded that a key SV binding site must be located within the missing 58 aa distal region of the C terminus.

### VISUALIZATION OF THE TETHER

The experiments above identify an SV binding site within the last 58 aa of the channel C-terminal. This terminal extends 641 aa from the cytoplasmic face of the transmembrane helix (aa 1718; *Mobyle predictor*). Secondary structure informatics (*Phyre*^2^ bioinformatics server) identified few alpha helices, beta sheets, or other ordered regions. Thus, allowing 0.36 nm/aa (the length of the polypeptide backbone) for disordered regions and 0.54 nm/3.6 aa’s for α-helices, 1/3rd of the disordered length for β-sheets (the length of these cannot be estimated with confidence but they were in any case minor) we can predict a maximum (limiting) length of up to 200 nm. Obviously other folding is possible and the terminal could be shorter but, nonetheless, this “back-of-the-envelope” calculation confirms that it is at least theoretically possible that an SV tethered to the end of the C-terminal could yet be located as far as ~4 SV diameters from its docking site within the AZ.

**FIGURE 3:**
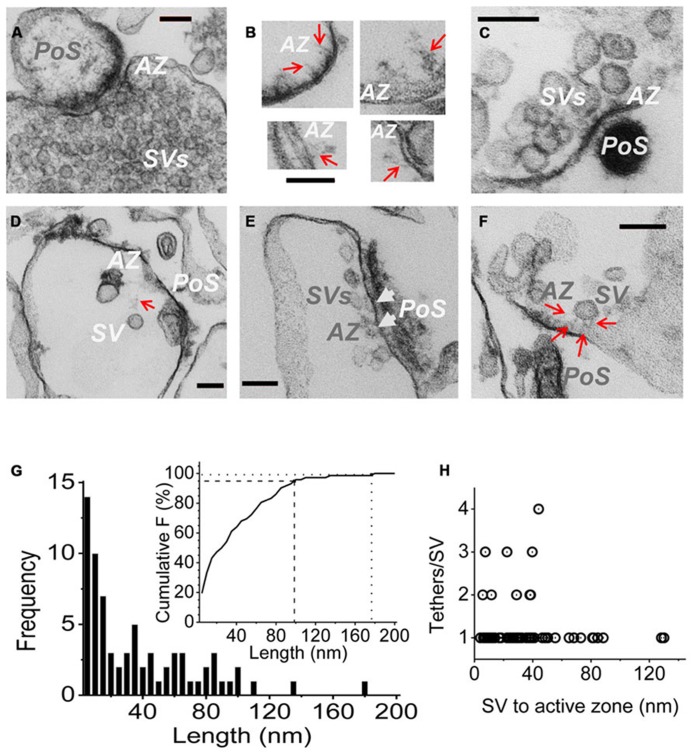
**Imaging SV tethers. (A–F)** Each panel shows an electron micrograph (100 nm section) of a chick brain synaptosome AZ, comprising the presynaptic terminal with its attached postsynaptic “scab” (PoS) or a structure that corresponds to a “condensed” scab (e.g., **C**). **(A)** is an SSM fixed prior to osmotic rupture showing a presynaptic C-terminal with dense cytoplasm and clouds of synaptic vesicles while **(B–F)** are EMs of “SSM ghosts” in which the cytoplasm was discharged by osmotic rupture prior to fixation (buffer Ca^2^^+^ clamped at 0.1 μM). **(B)** Four examples of fibrous material extending from the AZ (*red arrows*). **(C)** AZ with a cluster of retained SVs showing short fibrous extensions in the AZ region. **(D)** AZ with a single remote SV and a faint but distinct fibrous connection. **(E)** Extensive AZ with tethered close SVs. SVs presumed to be docked are attached to the surface membrane and indicated by *white arrow heads*. **(F)** AZ with a single close (~30 nm) SV clearly showing multiple fibrous attachments. AZ, active zone; SV, synaptic vesicle. Scale bars are 100 nm. **(G)** Frequency histogram of tether lengths measured from the leading edge of the SV to the AZ membrane along the tether when visible, and directly where the SV was too close to the surface membrane to resolve tethers. *N *= 72. *Inset*. Cumulative frequency histogram of tether lengths with 95 (dashed line) and 99% (dotted line) confidence limits, corresponding to 98 and 176 nm, respectively, indicated. **(H)** Plot of number of tethers versus tether length for each SV. Note, up to ~45 nm the SVs are tethered by 1–4 visible links but more distant SVs only exhibit one tether.

To explore this prediction we set out to image tethered SVs by transmission EM. While such connections have been imaged by cryo-electron tomography, this is a technically and computationally intensive method (Siksou et al., 2007; Fernandez-Busnadiego et al., 2010) and we sought a simpler approach. It is not possible in a standard EM to distinguish tethered SVs from their non-tethered neighbors within the dense cytoplasm (e.g., **Figure 3A**). However, we reasoned that if the untethered SVs and other contents of the SSM could be passively discharged, SVs that were physically attached to the surface membrane should remain. Such a model was at hand: SSM rupture and content-discharge, achieved by osmotic shock, is a routine step in our SV isolation protocol. We isolated the resulting nerve terminal membranes, “SSM ghosts” (Whittaker, 1968), using a discontinuous sucrose gradient (see Materials and Methods) and imaged these by EM. Consistent with previous reports, most SSMs had lost their intracellular components but some retained a few organelles including mitochondria, endoplasmic reticulum and, as anticipated, SVs.

Active zones were identified primarily by two standard criteria: darkening of the surface membrane and by the residual “scab” of postsynaptic apparatus that frequently remains attached (Whittaker, 1968; Nath et al., 2014). Fuzzy fibrous extensions of varying length and complexity were frequently observed (**Figure 3B**; Landis et al., 1988). Residual SVs were located at varying distances from the AZ (**Figures 3C–F**), ranging from intimately attached, and presumably docked (**Figure 3E**, *arrow heads*) to relatively remote within the surrounding cytoplasm (**Figure 3D**). By far the majority (>70%) of the cytoplasmic SVs in the AZ region could be seen to be linked to the AZ via fibrous processes, defined as morphological tethers (**Figures 3C–F**). We measured the length and number of SV tethers, tracing the course of the fiber to the AZ or, if the SV was close to the surface membrane so that connections could not be seen, we simply measured the inter-membrane distance (**Figure 3G**). A cumulative frequency histograms plot (**Figure 3G**, inset) indicates that 95% of these morphological tethers are up to 100 nm long and 99% are up to 175 nm, which we take as our estimate of the upper limit of the morphological tether lengths. Interestingly, a plot of the number of tethers against length indicated that the more distant SVs were linked to the AZ by a single tether (**Figure 3D**) whereas SVs that were closer, less than ~45 nm (e.g., **Figure 3F**), often exhibited multiple links (**Figure 3H**).

### INTERACTION BETWEEN RIM AND THE C-TERMINAL

Thus far our results were not inconsistent with published models of C-terminal/SV tethering. We next set out to test SV binding to the distal C-terminal and to explore the role of RIM. As noted above, RIM was pulled down by C3_Strep_ from SSM lysate (**Figure 1B**) which contains both reported RIM attachment sites (Kaeser et al., 2011): the PDZ DDWC and a more proximal P^*^^*^P domain, **Figure 1A**) domain associated with RBP binding. However, C3_Prox_ which includes the P^**^P domain, but lacks the terminus PDZ ligand domain (**Figure 1A**), failed to capture RIM (**Figure 1B**; SV lysate analysis 0.40 ± 0.10 *U*, *N* = 5, p_t_ = 0 < 0.02). These results argue that RIM does not bind to the C3 region via the P^**^P site in our assay (see also below).

### MUTANT FUSION PROTEIN ANALYSIS OF PDZ LIGAND DOMAIN BINDING

To explore the role of the channel PDZ ligand domain in SV tethering we next created two additional fusion proteins: C3_WildF_ and C3_MutantF_ (**Figure 1A**). C3_WildF_ is identical to the native C3_Strep_ but has an added distal C terminus FLAG tag, a mutation that can be predicted to eliminate PDZ ligand activity (Giallourakis et al., 2006; Tonikian et al., 2008). C3_MutantF_ is the same as C3_WildF_ but to rule out the remote possibility that the PDZ ligand domain remains functional even when displaced from the terminus (see Lee and Zheng, 2010), we created a second fusion protein in which we also mutated the DDWC sequence to DRYG.

**FIGURE 4:**
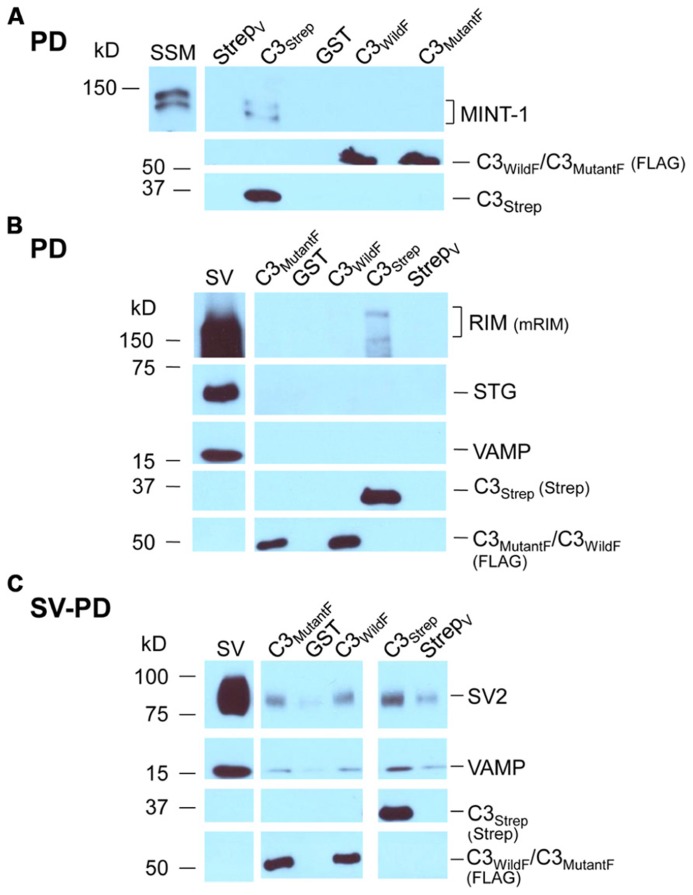
**Mutant PDZ ligand domain C-terminals and SV capture. (A)** C3_Strep_, but not C3_WildF_ or C3_MutantF_, pulls down MINT-1 from SSM lysate. **(B)** C3_Strep_, C3_WildF_ or C3_MutantF_ fail to pull down the integral SV proteins STG and VAMP from solubilized SV lysate while only C3_Strep_ captures RIM. **(C)** C3_Strep_, C3_WildF_ and C3_MutantF_ capture SVs from a suspension in a detergent-free buffer (HB), as indicated by two proteins, SV2 and VAMP. The positive control of SV-PD with C3_Strep_ is shown to the right. The use of different antibodies to identify the fusion proteins, anti-FLAG and anti-Strep, respectively, precluded quantitative comparison.

The channel C-terminal PDZ ligand domain was originally identified and characterized by its binding to the adaptor protein, MINT-1 (Maximov et al., 1999). We used this property to characterize our C-terminal fusion proteins. Thus, C3_Strep_, with a normal C-terminus, reliably captured MINT-1 from chick SSM lysates (Gardezi et al., 2013) in contrast to either C3_WildF_ (*N *= 3) or C3_MutantF_ (*N *= 3; **Figure 4A**). We also noted weak, but detectable RIM pull down with C3_Strep_ from SV lysate (**Figure 4B**) consistent with binding of this protein to the PDZ ligand domain (Kaeser et al., 2011).

Standard pull down experiments were also used to test C3 mutants for PDZ-dependent RIM binding. As above, the fusion proteins were immobilized and incubated with solubilized SVs and RIM capture was assessed by Western blot. In contrast to C3_Strep_, neither mutant recovered RIM (C3_WildF_ 0.002 ± (SE) 0.001 *U*, *N* = 3, *p* = 0.367; C3_MutantF_ 0.03 ± 0.02 *U*, *N* = 4, *p* > 0.1; **Figure 4B**), providing compelling support for the idea that the C-terminal can bind to RIM via a PDZ interaction (Kaeser et al., 2011). 

### MUTATION OF THE PDZ DOMAIN DOES NOT BLOCK SV CAPTURE

Thus far we confirmed that the normal distal C-terminal C3 region can capture SVs and that RIM binds to the PDZ ligand domain. We predicted that if RIM is critical for SV tethering the mutant C3 fusion proteins should fail to capture SVs. To our surprise, as assessed by SV-PD, both C3_WildF_ and C3_MutantF_ captured SVs with probabilities that were not significantly different from C3_Strep_ (**Figure 4C**; **Tables 2A,B**). Further, SV capture by the two mutants was greater than with C3_Prox_ using a similar binomial analysis, even with the relatively low *N* values of the latter.

### MIMETIC C3 PDZ DOMAIN PEPTIDES BLOCK PDZ LIGAND BINDING

**FIGURE 5:**
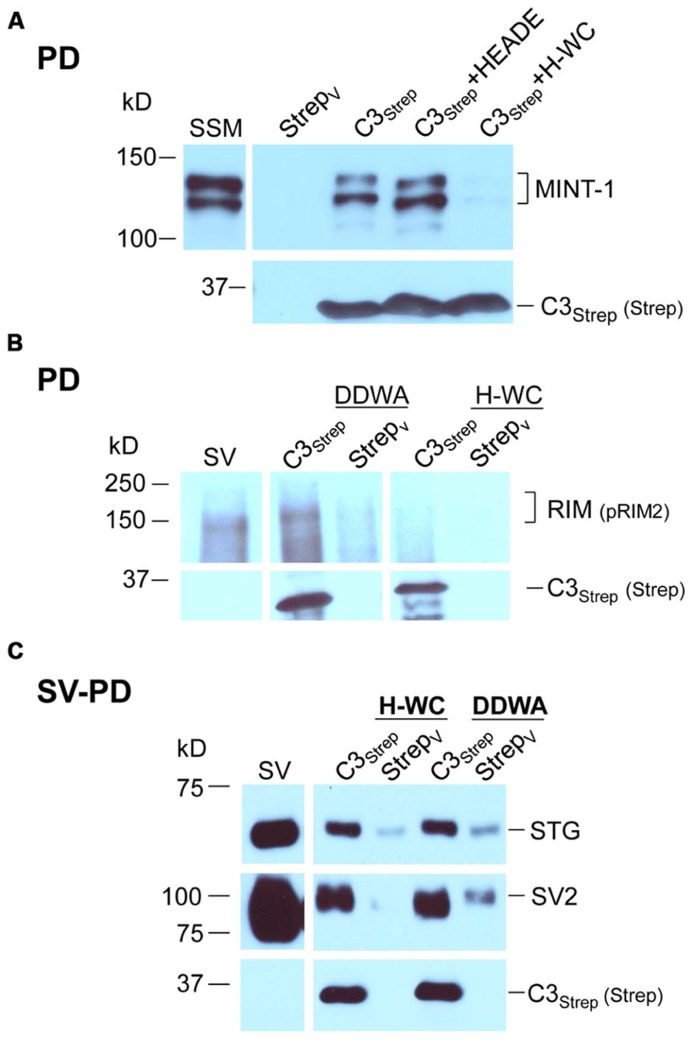
**The C-terminal PDZ ligand domain blocking mimetic peptide, H-WC, does not block SV capture by the C-terminal. (A)** SSM lysate was incubated with H-WC (HEADEDDWC) or HEADE (control, corresponding to the proximal, non-PDZ ligand domain, region of H-WC) peptides followed by pull-down with C3_Strep_ fusion protein and Western blot analysis of captured MINT-1. In the absence of peptide or with HEADE, C3_Strep_ successfully pulls down MINT-1 but this is markedly inhibited in the presence of H-WC. *N *= 4 comparing HEADE and H-WC (whole SSM or SSM membrane lysate). **(B)** RIM is pulled down from SV lysates by C3_Strep_ alone or in the presence of control peptide (DDWA) but is markedly inhibited by H-WC. **(C)** SV-PD persists in the presence of H-WC, PDZ ligand domain-blocking peptide, as indicated by capture of STG and SV2. DDWA served as a control.

The mutant fusion protein approach provides compelling evidence that SVs can bind to the distal C-terminal and that this can occur via a PDZ-RIM-independent mechanism. However, there was a remote possibility that the modified fusion proteins might snare SVs by an anomalous mechanism. We therefore devised a complimentary but independent analysis based on mimetic blocking peptides. Initial tests with a short four aa C-terminus peptide of the PDZ ligand domain alone, DDWC (aa 2354–2357), were abandoned due to non-specific blocking effects on unrelated proteins. However, as tested using SSM lysates, a longer nine aa C terminus mimetic peptide, HEADEDDWC (H-WC, aa 2349–2357), selectively inhibited MINT-1 pull down with C3_Strep_ (*N = *6; **Figure 5A**). In contrast, control experiments using H-WC pre-PDZ-ligand domain region HEADE (**Figure 5A**), an inactivated PDZ ligand domain peptide DDWA (with a mutated terminus cysteine), or the P^**^P-domain mimetic peptide RQLPQTPL, had little effect (**Table 2B**). Thus, H-WC was an effective and selective competitive blocker for the channel C-terminal PDZ ligand domain. Further, the lack of effect of the P^**^P domain peptide provided additional evidence that the SVs are not captured via the RBP site.

### MIMETIC PDZ DOMAIN PEPTIDE BLOCKS C-TERMINAL RIM BINDING BUT NOT SV CAPTURE

Consistent with the MINT-1 result and its interaction by PDZ binding, H-WC inhibited RIM pull down by C3_Strep_ (*N = *3; **Figure 5B**). However, H-WC did not inhibit SV-PD with C3_Strep_ as assessed by SV marker protein capture (**Figure 5C**; **Tables 2A,B**). Thus, the blocking peptide results supported the conclusion that SVs can bind to the C-terminal *independently* of RIM or the PDZ domain.

### MIMETIC C3 PDZ DOMAIN AND RBP BINDING DOMAIN PEPTIDES DO NOT BLOCK SV TURNOVER IN FUNCTIONAL SSMs

If the above *in vitro* binding experiments accurately reflect the molecular basis of CaV2.2 C-terminal-based tethering, we can predict that in the intact nerve terminal depolarization-gated SV turnover should persist in the presence of PDZ ligand domain or P^**^P mimetic blocker peptides. This hypothesis was tested in the chick SSMs using our novel “cryoloading” method (Nath et al., 2014) that permits the introduction of large (up to at least 150 kD) membrane-impermeable alien compounds into functional SSMs. Briefly, fresh SSMs were frozen in a buffer containing the test compound, a 3 kD dextran-FITC loading marker and a cryoprotectant. Our findings suggest that extracellular medium is admitted by bulk transfer when the surface membrane cracks and reseals upon defrosting. Cryoloaded SSMs were functional and exhibited normal depolarization/Ca^2^^+^-dependent SV recycling, as assessed using fluorescent styryl dye uptake and release. The demonstration that dye uptake is blocked when the SSMs are cryoloaded with membrane-impermeant BAPTA or botulinum toxin A light chain (Bot A-LC) verifies the method (Nath et al., 2014).

**FIGURE 6:**
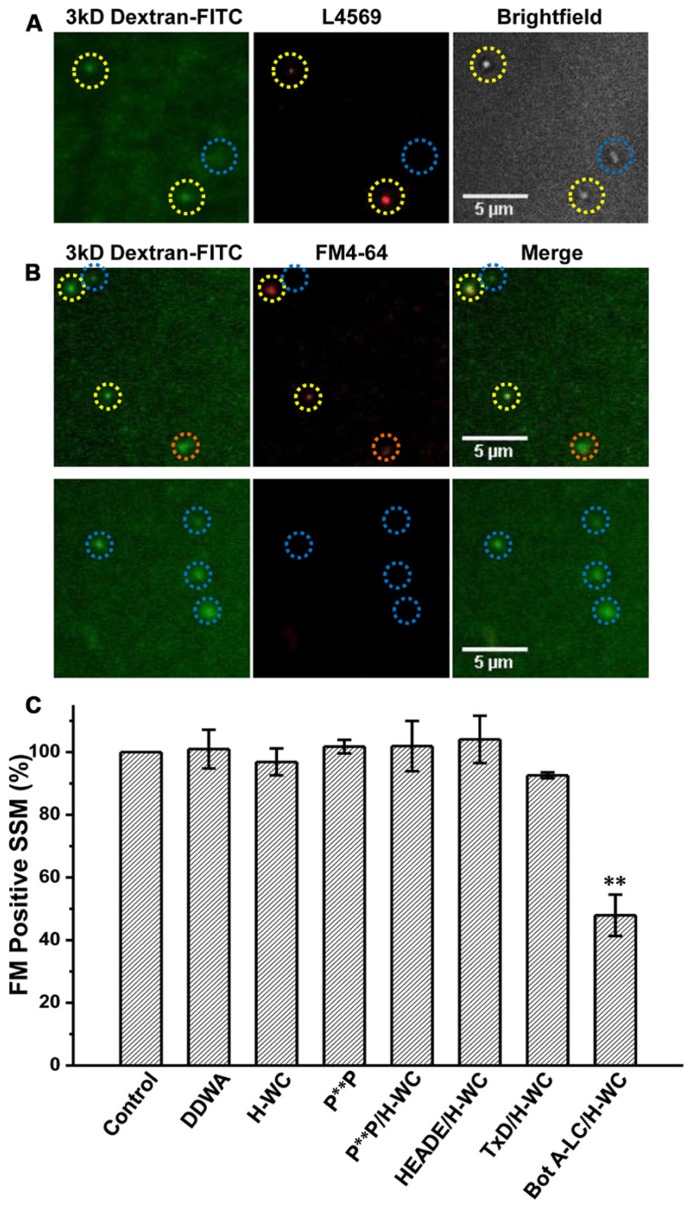
**H-WC PDZ ligand domain-blocking peptide does not affect presynaptic styryl dye recycling. (A)** SSMs were cryoloaded with H-WC peptide (1.2 mM) together with 3 kD Dextran-FITC 20 μM. The dextran marker (*left panel*) identifies SSMs (Nomarski bright field, *right panel*) that were cryoloaded with the peptide and was confirmed with an antibody raised against the C terminus (L4569; Khanna et al., 2006b; *red*, *center panel*). Images were taken with a fixed short shutter open time (300 ms) to avoid detection of intrinsic CaV2.2 channels. SSMs that were positive for both dextran and L4569 are indicated (*yellow circles*) while an SSM that failed to take up dextran was also negative for H-WC staining (*blue circle*). **(B,C)** The PDZ-ligand mimetic blocker H-WC does not block SV recycling in synaptosomes. SSMs were cryoloaded with the indicated test compounds as in **(A)**. The defrosted terminals were depolarized with elevated K^+^ (40mM) in the presence of Ca^2^^+^ (1.2 mM) to trigger exocytosis and uptake of FM4-64 by membrane recycling. The fraction of FM-stained terminals was normalized to a paired dextran-only control experiment (see Nath et al., 2014). **(B)** Upper panel. SSMs loaded with H-WC alone (with carrier buffer as for Bot A-LC). Lower panel. SSMs loaded with H-WC, as in **(A)**, together with Bot A-LC. Dashed circles indicate cryoloaded (dextran positive) SSMs that exhibit strong (*yellow*), moderate (*orange*) or no (*blue*) FM uptake. **(C)** Histograms of percent ± SE of dextran-positive, and hence cryoloaded terminals that were FM4-64 positive for three separate experiments. Cryoloaded compound(s), concentration and statistical test to the dextran-only *Control* were: *DDWA* (1.2 mM), *p* > 0.1; *H-WC* (1.2 mM), *p* > 0.1; P^**P^ (1.2 mM), *p* > 0.1; P**P/H-WC (1.2 mM each), *p* > 0.1; *HEADE/H-WC* (1.2 mM each) *p* > 0.1; *TxD/H-WC* tetanus toxoid (200 nM and 1.2 mM, respectively) *p* > 0.1. *BotA-LC/H-WC* (200 nM and 1.2 mM, respectively) was significantly different from *Control* *p* < 0.05, *H-WC* (*p* < 0.01), *SH3 *+ *H-WC* (*p* < 0.01), and *TxD *+ *HDWC* (*p* < 0.05).

Synaptosomes were cryoloaded with the PDZ ligand domain and P^**^P domain mimetic peptides, H-WC and RQLPQTPL, respectively, individually or both together using DDWA as a peptide loading control and fluorescent dextran (3 kD) to mark cryoloaded SSMs. Uptake of H-WC into the SSMs was confirmed by two methods. First, the peptide was detected in the cryoloaded, but not the unloaded SSMs on the same plate by immunostaining using our anti-long-splice distal C-terminal antibody, L4569 (Khanna et al., 2006b), combined with short-exposure fluorescence imaging (to minimize detection of intrinsic CaV2.2 channels; **Figure 6A**). SSMs cryoloaded with H-WC alone or with inactive botulinum toxoid (**Figure 6B**, *upper panel*) exhibited depolarization/Ca^2^^+^ styryl dye uptake (FM4-64) uptake as observed in controls. In contrast, a large fraction of SSMs that were cryoloaded with H-WC spiked with Bot A-LC [which is membrane impermeant; (see Nath et al., 2014)] failed to take up the styryl dye; **Figure 6B**, *lower panel*, an experiment that served as a positive control. The fraction of SSMs that recycled SVs, as assessed by styryl dye uptake, was not reduced by any of these test peptides, whether cryoloaded alone or in combination (**Figure 6C**). Hence, neither PDZ ligand domain, nor P^**^P domain mimetic peptides exhibited inhibition of brain nerve terminal SV turnover.

## DISCUSSION

The main implications of this study are that SVs can tether to a novel site located in the distal CaV2.2 C-terminal but proximal to the HEADEDDWC tip. The project was made possible by the development of three novel methods: SV-PD to assay binding of SVs to calcium channel regions *in vitro* (Wong et al., 2013); SSM ghost EM to image tethered SVs at nanometer resolution and, cryoloading, a simple method to introduce peptide blockers into functional SSMs (Nath et al., 2014). We recently used the SV-PD method to demonstrate that presynaptic CaV2.2 channels can capture SVs *in vitro*, confirming that SVs can tether to the channel independently of the surface membrane and demonstrating direct binding to the C-terminal distal third using the C3_Strep_ fusion protein (Wong et al., 2013).

The failure of C3_Prox_ to capture SVs localizes the distal C-terminal binding site to the terminal 58 aa. A crude estimate of C-terminal length predicts that the SVs could be tethered by this mechanism as far as ~200 nm from the AZs (see also Wong et al., 2013). We used the SSM ghost EM method to image AZs after removal of the nerve terminal cytoplasm. Fibrous projections were observed and some of these exhibited attached SVs. Our cumulative histogram indicates that 95% of these morphological tethers are up to ~100 nm long and 99% are up to ~175 nm and, hence, within the estimated maximum length of the C-terminal. Structures linking the SV to the AZ have been imaged previously (Siksou et al., 2007; Fernandez-Busnadiego et al., 2010; Szule et al., 2012) and length estimates are consistent with our analysis (Siksou et al., 2007; Fernandez-Busnadiego et al., 2013). A second similarity was that SVs that are close to the surface membrane exhibit multiple links whereas only single tethers – presumed channel C-terminals – are observed for the more distant (>~45 nm) SVs [**Figure 3H**; see also (Fernandez-Busnadiego et al., 2013)]. This is particularly significant with respect to the mechanics of SV capture since it would be difficult to imagine how an SV would be captured and withdrawn for docking if it was attached to two or more remote CaVs at the same time. Since standard EM involves considerable tissue processing these results need to be reproduced using other super-resolution methods.

The idea that SVs are linked to the channel via its C-terminal was proposed by Kaeser et al. (2011) and our results provide broad support. However, they also concluded that the “SV tethers the channel” via RIM through two C-terminal connections: one directly to the PDZ ligand domain, and the other indirectly via RBP and the P^**^P site. Contrary to expectations, our *in vitro* assay failed to support either link. Several findings argued against a significant contribution of the RBP/P^**^P mechanism. We did not observe pull down of RIM with three different PDZ ligand domain-lacking fusion proteins that retained the P^**^P domain (C3_Prox_, **Figure 1B**; C3_WildF_ or C3_MutantF_, **Figure 4B**). This argues against RIM linking to the C-terminal via RBP. Lastly, we did not detect any inhibition of SV recycling *in situ* when terminals were loaded with a mimetic P^**^P-site blocking peptide (**Figure 6C**), a peptide that also had no detectable effect on SV-PD by the normal C3 fusion protein C3_Strep_ (**Table 2B**). These results suggest that marked changes in transmitter release physiology observed to occur after deletion of RBP (Liu et al., 2011) reflect a presynaptic defect unrelated to CaV distal C-terminal SV tethering.

We next explored the hypothesis that the channel C-terminal binds to the SV via a PDZ-interaction through RIM. The presence of a PDZ ligand domain on the tip of the C-terminal is well established (Maximov et al., 1999; Maximov and Bezprozvanny, 2002). The persistence of SV-PD with the PDZ mutant fusion proteins, C3_WildF_ and C3_MutantF_ indicates that the SV can interact with the C-terminal independently of the PDZ ligand domain. This result did not, however, rule out the possibility that there are two independent binding sites in the distal 58 aa, the PDZ ligand and also an additional site. To test the issue we created a mimetic blocking peptide of the C-terminus nine aa mimetic blocking peptide, H-WC. This peptide effectively inhibited the PDZ-ligand domain by marked inhibition of both MINT-1 and RIM pull down. However, H-WC failed to inhibit SV-PD by C3_Strep_ and hence, we were compelled to conclude that *in vitro* binding of SVs to the C-terminal distal region occurs via a novel, as yet uncharacterized site. This idea is consistent with a recent cryo-electron tomography analysis of SV tethering in RIM knockout mice (Fernandez-Busnadiego et al., 2013). Interestingly, the knockout terminals exhibited a marked deficit in SVs with the multi-short, but *not* the uni-long tethers! Thus, our biochemical analysis and this published ultrastructural one are mutually supportive.

The question then was whether the biochemical analysis accurately reflects the biology of SV tethering *in vivo*. To test this in normal CNS presynaptic terminals we first invented the cryoloading method to introduce alien compounds into SSMs (Nath et al., 2014). This was combined with a standard styryl dye assay of depolarization/Ca^2^^+^-dependent SV recycling. We have previously shown that cryoloaded Bot A-LC markedly reduces the fraction of SSMs that recycle SVs. Cryoloading of H-WC together with Bot A-LC exhibited a similar reduction and serves as a positive control. However, cryoloading H-WC alone (or with the P^**^P mimetic peptide) had no detectable effect on SV recycling, as monitored by FM uptake, supporting the above conclusion and arguing against a critical role for the PDZ-ligand domain in SV turnover. As for other channels (Piserchio et al., 2006), the primary function of the CaV2.2 PDZ-ligand domain may be for transport, targeting and channel release site scaffolding (Maximov et al., 1999; Maximov and Bezprozvanny, 2002; Han et al., 2011; Kaeser et al., 2011; Gardezi et al., 2013). Thus, we were unable to support the idea that SVs tether to the channel via a RIM/RBP/Rab link via the channel C-terminal P^**^P or PDZ ligand domain binding sites.

**FIGURE 7:**
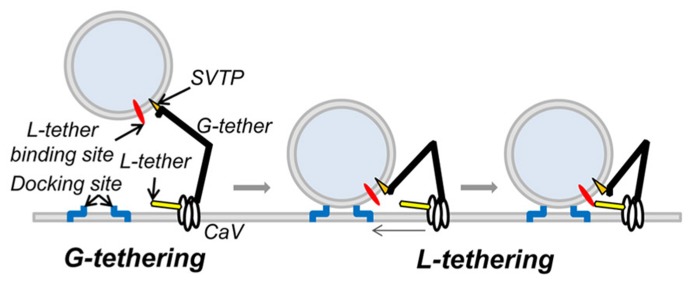
**Working model of CaV tethering of SVs at the transmitter release site.** Our suggest that the SV is initially tethered by binding to a site just proximal to the tip of the channel C-terminal. We hypothesize that this serves to grab, or “G-tether,” an SV from the cytoplasm in the AZ region and that an unknown mechanism then draws the SV into its docking site near the channel. However, since previous results suggest that the calcium sensor is within 25 nm of the channel mouth, we also hypothesize one or more additional CaV-SV links serves to lock or “L-tether” the SV within range of the calcium channel Ca^2^^+^ domain.

These findings implicate a novel SV tether site within the last 49 aa of the CaV2.2, proximal to the H-WC terminus. The idea that SVs are tethered to the calcium channel was predicted from the finding that SV fusion can be triggered by the opening of a single calcium channel (Stanley, 1993). For the SV calcium sensor to be exposed to a sufficiently high Ca^2^^+^ concentration it has to be located very close – within ~25 nm – from the channel pore (Stanley, 1997; Weber et al., 2010). While C-terminal tethering could account for the initial capture of SVs, it cannot readily explain the intimate relationship required for nanodomain gating. Thus, it would seem necessary to predict two tethering mechanisms: first, a remote one that “grabs” or *G-tethers* the SV from the cytoplasm, which may be accounted for by the C-terminal attachment identified above and a second, short-range one (Stanley, 1993; Fernandez-Busnadiego et al., 2010; Harlow et al., 2013), to “lock,” or *L-tether*, the SV within the channel Ca^2^^+^ domain (**Figure 7**). The finding that there are a reduced number of short-range tethers in RIM knockouts (Fernandez-Busnadiego et al., 2013) raises the possibility that this protein is involved in the latter, L-tethering, mechanism.

Our model of C-terminal-based, G-tethering begs two immediate questions. First, how is the SV retracted to dock after binding to the distal C-terminal? Previous studies have identified motor proteins at the release site (Mochida et al., 1994; Khanna et al., 2007; Kisiel et al., 2011) that could conceivably participate in G tether retraction, while numerous studies have postulated shorter range, putative L-tether links, in particular via the channel II–III loop (O’Connor et al., 1993; Sheng et al., 1994, 1996; Catterall, 1999; Atlas, 2001) that may work in series. The second critical question is the identity of the CaV2.2 distal C-terminal SV tether-attachment (SVTP) protein(s). At this point we have no idea. However, the observation that more distant SVs are tethered by a single link (**Figure 3H**; Fernandez-Busnadiego et al., 2013) suggests that the SV expresses only one, or at most a very few corresponding attachment sites. This inference is attractive mechanistically since it would ensure that an SV can only be recovered by one calcium channel at a time.

## Conflict of Interest Statement

The authors declare that the research was conducted in the absence of any commercial or financial relationships that could be construed as a potential conflict of interest.
